# Within-pair differences of DNA methylation levels between monozygotic twins are different between male and female pairs

**DOI:** 10.1186/s12920-016-0217-2

**Published:** 2016-08-26

**Authors:** Mikio Watanabe, Chika Honda, The Osaka Twin Research Group, Yoshinori Iwatani, Shiro Yorifuji, Hiroyasu Iso, Kei Kamide, Jun Hatazawa, Shinji Kihara, Norio Sakai, Hiroko Watanabe, Kiyoko Makimoto, Mikio Watanabe, Chika Honda, Yoshinori Iwatani

**Affiliations:** 1 Department of Biomedical Informatics, Osaka University Graduate School of Medicine, Yamadaoka 1-7, Suita, Osaka 565-0871 Japan; 2 Center for Twin Research, Osaka University Graduate School of Medicine, Yamadaoka 1-7, Suita, 565-0871 Osaka Japan

**Keywords:** Methylation, Monozygotic twin, Individual difference, Epigenetic change

## Abstract

**Background:**

DNA methylation levels will be important for detection of epigenetic effects. However, there are few reports showing sex-related differences in the sensitivity to DNA methylation. To evaluate their sex-related individual differences in the sensitivity to methylation rigorously, we performed a systematic analysis of DNA methylation in monozygotic twins, an optimal model to evaluate them because the genetic backgrounds are the same.

**Results:**

We examined 30 male and 43 female older monozygotic twin pairs recruited from the registry established by the Center for Twin Research, Osaka University. Their methylation levels were determined using the Infinium HumanMethylation450 BeadChip Kit (Illumina), which interrogated 485577 highly informative CpG sites at the single-nucleotide resolution, and the median methylation level was calculated for each of the 25657 CpG islands. Within-pair differences of methylation levels (WPDMs) were greater in male pairs than female pairs for 86.0 % of autosomal CpG islands, but were higher in female pairs than male pairs for 76.7 % of X chromosomal CpG islands. Mean WPDMs of CpG islands in each autosomal chromosome were significantly higher in male pairs than in female whereas that in X chromosome was significantly higher in female pairs than in male. Multiple comparison indicated that WPDMs in three autosomal and two X-chromosomal CpG islands were significantly greater in male pairs, whereas those in 22 X-chromosomal CpG islands were significantly greater in female pairs.

**Conclusion:**

Sex-related differences were present in the WPDMs of CpG islands in individuals with the same genetic background. These differences may be associated with the sexual influences in susceptibility of some diseases.

**Electronic supplementary material:**

The online version of this article (doi:10.1186/s12920-016-0217-2) contains supplementary material, which is available to authorized users.

## Background

Human phenotypes, such as physical characteristics, abilities, and disease susceptibility, are determined by both genetic and environmental factors [1–4]. Environmental factors affect human phenotypes by changing the epigenetic modification of the genome, such as by DNA methylation and histone modification [5]. Epigenetic modification changes impact cellular behavior by regulating the chromatin status and gene expression [6] and so the evaluation of epigenetic changes will be used as new laboratory tests. One of the most important epigenomic modifications is the methylation of genomic DNA, which is the covalent addition of a methyl group to the cytosine at CpG dinucleotides. The CpG sites present in the regions containing high numbers of CpG dinucleotides (CpG islands) are generally unmethylated, although those in the majority of other genomic regions are methylated. CpG islands overlap the promoter regions of 60–70 % of genes and are generally protected from methylation, allowing for the expression of downstream genes, the transcription of which is further regulated by histone modification [7].

Many reports show the within-pair differences of methylation levels (WPDMs) in discordant monozygotic twins for several disorders and traits because the aberrant DNA methylation of CpG islands may be an important epigenetic change that affects the developmental process of diseases or traits [8–19]. To identify the association of DNA methylation with the development of disease, general WPDMs in monozygotic twin pairs should be assessed. However, they have not yet been elucidated.

In this study, we examined the methylation levels of CpG islands in 113 monozygotic twins, calculated the WPDMs of genomic DNA, and compared the WPDMs between men and women to identify the sex difference in the WPDMs. WPDM of monozygotic twins can reflect the difference of the sensitivity to DNA methylation under the condition of the same genetic background. This study will be able to clarify the sex-related differences in the sensitivity to DNA methylation.

## Subjects and Methods

### Subjects

A total of 113 healthy Japanese monozygotic twin volunteers (35 male and 78 female pairs) were recruited from the registry established by the Center for Twin Research, Osaka University (Table 1) [20]. Blood was sampled at 9 am after a 12 h fast. A clinical examination was performed, and the twins completed health-related questionnaires. The twins in each pair were examined on the same day. Genomic DNA was isolated from peripheral blood mononuclear cells using a commercial kit (QIAamp DNA Mini Kit, QIAGEN, Germany). The zygosity of subjects was confirmed by the perfect matching of 15 short tandem repeat (STR) loci using the PowerPlex® 16 System (Promega, Madison, WI, USA).

**Table 1:** Character of examined twins

			Gender	
			Male	Female
all twins	n (pair)		35	78
	age			
		(mean ± SD)	67.4 ± 15.0	55.5 ± 17.0
		(range)	22–87	21–87
elder	n (pair)		30	43
subset	age			
		(mean ± SD)	71.8 ± 9.6	68.1 ± 8.6
		(range)	57–87	55–87

### Methylation level of CpG islands

Analysis of the methylation level was performed using an Infinium HumanMethylation450 BeadChip Kit (Illumina), which interrogated 485577 highly informative CpG sites at the single-nucleotide resolution for each sample using the standard manufacturer's protocol. The experiment was performed with 0.5 μg of high-quality genomic DNA. There were 2 bead types for each CpG site per locus on the chip. The raw data were analyzed using the Genome Studio software (Illumina), and the fluorescence intensity ratios between the 2 bead types were calculated. A ratio value of 0 was equal to the nonmethylation of the locus, and a ratio of 1 was equal to total methylation. These raw data were corrected to normalize the differences in detection ranges between the two probes of the Infinium Assay using a peak-based correction method [21]. Normalized data were filtered to exclude invalid probes, such as null probes and probes with low reliability. After filtering, the data were categorized to each of 25657 CpG islands according to the registration of UCSC [22, 23], and a median methylation level was calculated when there were two or more probes in a CpG island. We used the statistical software R (ver.2.15.1) to perform these data analyses.

### Within-pair differences of the methylation level (WPDM)

We calculated the absolute values of differences in each CpG island methylation level between individuals in each pair as follows:

$$ WPDM = \left|\  ML1 - ML2\ \right| $$

where ML1 is the methylation level of one of each twin pair and ML2 is that of the other twin.

We also calculated the gender difference index of WPDMs in each CpG island as follows

$$ Gender\  difference\  index = mean\  of\  male\  WPDMs - mean\  of\  female\  WPDMs $$

This index is positive when the mean WPDM of a CpG island is higher in a male pair than a female pair.

### Statistical analysis

Student’s *t* test was used to compare WPDMs between males and females. Statistical analysis was performed using the JMP10 software (SAS Institute, Inc., Tokyo, Japan).

## Results

### Within-pair differences in the methylation levels (WPDMs) of CpG islands

**Fig. 1:**
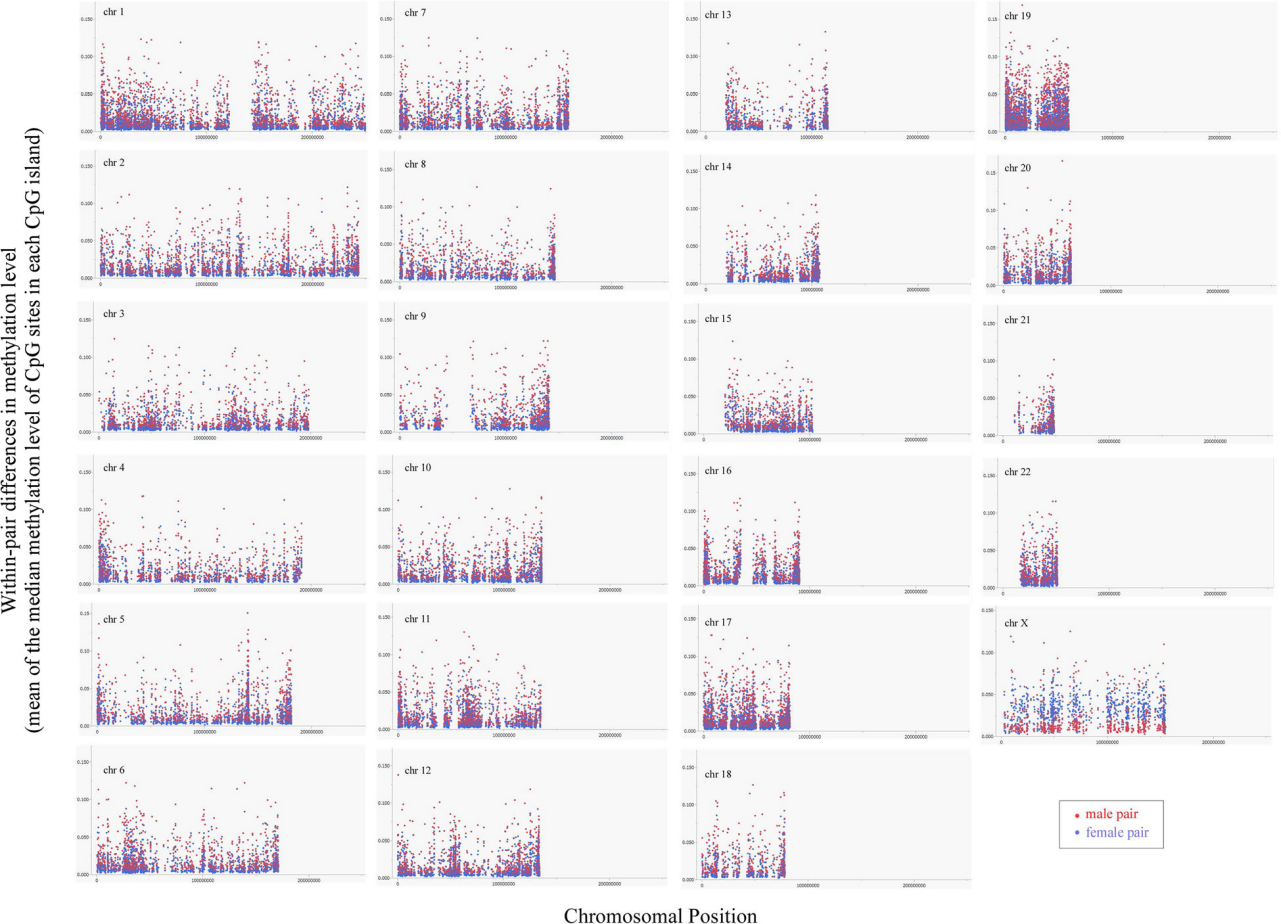
Within-pair differences in methylation levels for each CpG island (older pairs). Red circles indicate male pairs, and blue circles indicate female pairs. Within-pair differences in older male pairs are also greater in most autosomal CpG islands

As shown in Additional file 1: Figure S1, we could find that the WPDMs were larger in many autosomal CpG islands for male pairs than female pairs, whereas the WPDM in many X chromosomal CpG islands were larger in female pairs than male pairs. When we performed the same analysis using only an older subset (>55 years old) (Table 1), we obtained similar results (Fig. 1). As shown in Table 2, means WPDM of CpG islands in each autosomal chromosome were significantly higher in male than in female pairs, whereas that in X chromosome was significantly higher in female than in male pairs. In addition, median of WPDM were also showed the same significances (Table 2).

**Table 2:** WPDMs of CpG islands in each chromosomes

Chromosome	number of analyzed CpG island	Mean ± SD of WPDM			Median (range) of WPDM		
		Male	Female	*P* value (student's *t* test)	Male	Female	*P* value (MannWhitney test)
1	2327	**0.014** ± **0.013**	0.009 ± 0.009	1.17 × 10^–47^	**0.009** (**0.002**–**0.093)**	0.005 (0.0005–0.067)	0
2	1618	**0.015 ± 0.013**	0.010 ± 0.009	7.42 × 10^–43^	**0.010 (0.002–0.079)**	0.006 (0.0009–0.061)	0
3	1132	**0.013 ± 0.011**	0.008 ± 0.009	2.18 × 10^–28^	**0.008 (0.002–0.086)**	0.005 (0.0006–0.077)	0
4	982	**0.015 ± 0.013**	0.010 ± 0.009	1.40 × 10^–24^	**0.011 (0.002–0.080)**	0.008 (0.0008–0.055)	0
5	1177	**0.016 ± 0.014**	0.011 ± 0.010	1.37 × 10^–27^	**0.011 (0.002–0.093)**	0.007 (0.0006–0.073)	0
6	1220	**0.015 ± 0.013**	0.009 ± 0.009	3.13 × 10^–30^	**0.010 (0.002–0.113)**	0.006 (0.0004–0.066)	0
7	1460	**0.015 ± 0.013**	0.010 ± 0.009	3.27 × 10^–30^	**0.010 (0.001–0.090)**	0.007 (0.0010–0.063)	0
8	959	**0.015 ± 0.013**	0.010 ± 0.009	1.30 × 10^–22^	**0.010 (0.002–0.075)**	0.007 (0.0007–0.070)	0
9	786	**0.016 ± 0.014**	0.009 ± 0.009	1.75 × 10^–35^	**0.011 (0.002–0.080)**	0.006 (0.0011–0.052)	0
10	1092	**0.016 ± 0.013**	0.010 ± 0.009	1.95 × 10^–26^	**0.010 (0.002–0.079)**	0.007 (0.0007–0.058)	0
11	1343	**0.014 ± 0.013**	0.010 ± 0.009	9.44 × 10^–26^	**0.009 (0.001–0.082)**	0.006 (0.0007–0.062)	0
12	1185	**0.014 ± 0.012**	0.009 ± 0.009	3.99 × 10^–23^	**0.009 (0.002–0.080)**	0.006 (0.0010–0.061)	0
13	556	**0.016 ± 0.013**	0.011 ± 0.009	8.51 × 10^–13^	**0.010 (0.002–0.092)**	0.007 (0.0010–0.046)	1.60 × 10^–14^
14	742	**0.014 ± 0.013**	0.009 ± 0.008	8.36 × 10^–21^	**0.009 (0.001–0.083)**	0.006 (0.0008–0.070)	0
15	725	**0.014 ± 0.012**	0.009 ± 0.008	1.61 × 10^–20^	**0.009 (0.002–0.075)**	0.006 (0.0009–0.055)	0
16	1363	**0.014 ± 0.012**	0.010 ± 0.009	2.72 × 10^–24^	**0.010 (0.002–0.096)**	0.008 (0.0008–0.056)	0
17	1558	**0.014 ± 0.012**	0.009 ± 0.009	3.91 × 10^–28^	**0.009 (0.002–0.082)**	0.006 (0.0007–0.064)	0
18	487	**0.016 ± 0.014**	0.011 ± 0.010	7.45 × 10^–13^	**0.011 (0.002–0.103)**	0.008 (0.0009–0.078)	4.00 × 10^–15^
19	2441	**0.015 ± 0.013**	0.011 ± 0.010	4.56 × 10^–39^	**0.010 (0.002–0.087)**	0.007 (0.0008–0.081)	0
20	784	**0.016 ± 0.013**	0.011 ± 0.090	7.60 × 10^–19^	**0.011 (0.002–0.125)**	0.008 (0.0008–0.065)	0
21	334	**0.014 ± 0.011**	0.011 ± 0.009	1.64 × 10^–6^	**0.010 (0.002–0.085)**	0.008 (0.0013–0.052)	1.55 × 10^–8^
22	661	**0.014 ± 0.012**	0.010 ± 0.009	1.50 × 10^–10^	**0.010 (0.002–0.080)**	0.007 (0.0011–0.067)	3.77 × 10^–15^
X	725	0.015 ± 0.013	**0.022 ± 0.009**	0	0.010 (0.002–0.078)	**0.022 (0.0022–0.056)**	0

Boldface types indicate significanlty higher WPDM values

The WPDMs of CpG islands in older male and female pairs are shown in Additional file 2: Table S1 in ranking order. Table 3 shows the top-rank 50 CpG islands, which have large WPDMs in older male and female pairs, and the common CpG islands, which are included in the top-rank 50 CpG islands of both genders. These are shown in Table 4.

**Table 3:** Rank order within-pair differences in methylation levels of CpG islands in elder men and women pairs (Top-rank 50, in descending order)

Elder male pairs		Elder female pairs	
CpG Islands	MEAN ± SD	CpG Islands	MEAN ± SD
chr19:15833733–15833983	0.169 ± 0.171	chr3:128215212–128216905	0.108 ± 0.119
chr20:54824312–54824584	0.167 ± 0.150	chr7:138348962–138349444	0.107 ± 0.115
chr5:140255158–140255450	0.151 ± 0.150	chr19:15833733–15833983	0.104 ± 0.128
chr12:312591–313331	0.138 ± 0.134	chr20:54824312–54824584	0.101 ± 0.119
chr5:1494853–1495287	0.136 ± 0.129	chr4:74847528–74847830	0.098 ± 0.127
chr13:112627428–112627642	0.133 ± 0.117	chr11:67052394–67053110	0.097 ± 0.097
chr19:5074591–5074814	0.133 ± 0.118	chr17:70120139–70120442	0.095 ± 0.092
chr11:62314761–62315054	0.130 ± 0.115	chr19:5074591–5074814	0.094 ± 0.090
chr20:22567453–22567880	0.130 ± 0.129	chr18:77552401–77552603	0.092 ± 0.076
chr5:140764301–140764680	0.129 ± 0.148	chr8:43131177–43131487	0.092 ± 0.081
chr17:7492314–7492945	0.129 ± 0.125	chr17:80346597–80347050	0.092 ± 0.097
chr17:6797429–6797724	0.128 ± 0.135	chr1:149162389–149162615	0.090 ± 0.110
chr10:105428505–105428713	0.128 ± 0.124	chr19:4950670–4950940	0.090 ± 0.068
chr8:72753874–72754755	0.127 ± 0.128	chr4:40752691–40752896	0.090 ± 0.097
chr18:47825069–47825325	0.127 ± 0.154	chr19:39993357–39993765	0.089 ± 0.096
chrX:65041896–65042304	0.125 ± 0.134	chr2:208546082–208546562	0.089 ± 0.102
chr7:27134097–27134303	0.125 ± 0.122	chr8:1321232–1321638	0.089 ± 0.101
chr3:14597400–14597651	0.125 ± 0.141	chr19:57276614–57276942	0.088 ± 0.082
chr8:142219197–142219445	0.125 ± 0.120	chrX:70316349–70316671	0.088 ± 0.107
chr7:73118500–73118749	0.125 ± 0.114	chr6:27482888–27483089	0.088 ± 0.095
chr17:40700164–40700859	0.125 ± 0.141	chr22:25081850–25082112	0.088 ± 0.083
chr19:48047796–48049162	0.124 ± 0.122	chr18:13641584–13642415	0.086 ± 0.083
chr15:27212902–27213396	0.124 ± 0.120	chr22:27834425–27834630	0.086 ± 0.115
chr11:67052394–67053110	0.124 ± 0.112	chr2:131010510–131010764	0.085 ± 0.089
chr1:38200919–38201200	0.124 ± 0.123	chrX:139521561–139521897	0.085 ± 0.105
chr17:18575709–18576477	0.123 ± 0.125	chr18:74114551–74114791	0.085 ± 0.072
chr1:47899125–47899398	0.123 ± 0.118	chr7:57270847–57271464	0.084 ± 0.101
chr5:140221007–140221381	0.123 ± 0.117	chr12:125003217–125003482	0.084 ± 0.097
chr6:27482888–27483089	0.123 ± 0.100	chr6:139116946–139117469	0.084 ± 0.102
chr6:139116946–139117469	0.123 ± 0.112	chr10:101824961–101825186	0.084 ± 0.081
chr9:139715663–139716441	0.122 ± 0.116	chr13:112627428–112627642	0.083 ± 0.102
chr9:135361992–135362481	0.122 ± 0.133	chr3:99594969–99595215	0.083 ± 0.076
chr2:232526666–232527777	0.122 ± 0.125	chr1:156261199–156261425	0.082 ± 0.086
chr19:8397958–8400461	0.122 ± 0.121	chr2:157184389–157184632	0.082 ± 0.082
chr9:69500968–69501225	0.121 ± 0.149	chr1:2082314–2082529	0.082 ± 0.066
chr19:44860657–44860928	0.121 ± 0.128	chr19:21265164–21265433	0.082 ± 0.106
chr2:121279842–121280183	0.120 ± 0.120	chr5:140181888–140183014	0.082 ± 0.083
chr2:131186145–131186496	0.120 ± 0.129	chr9:137252115–137252451	0.082 ± 0.083
chr1:149162389–149162615	0.120 ± 0.121	chr9:135361992–135362481	0.081 ± 0.090
chr11:35965642–35966454	0.119 ± 0.103	chr4:174421347–174421559	0.081 ± 0.087
chr1:75590817–75591354	0.119 ± 0.122	chr13:88329394–88329885	0.081 ± 0.130
chrX:8751285–8751608	0.119 ± 0.138	chr4:74719087–74719339	0.080 ± 0.095
chr1:43472867–43473334	0.119 ± 0.113	chrX:40064743–40064993	0.080 ± 0.100
chr12:125003217–125003482	0.119 ± 0.112	chr6:170589411–170590085	0.079 ± 0.101
chr19:4059917–4060131	0.119 ± 0.115	chr1:75590817–75591354	0.079 ± 0.105
chr1:149230771–149231197	0.119 ± 0.130	chr22:46658397–46659332	0.079 ± 0.092
chr4:41749184–41749811	0.118 ± 0.098	chr15:31689500–31689707	0.079 ± 0.074
chr6:35754713–35754914	0.118 ± 0.130	chr3:151178623–151178984	0.079 ± 0.119
chr14:103604539–103605504	0.118 ± 0.121	chr19:940723–942490	0.079 ± 0.069
chr1:240656253–240656720	0.118 ± 0.123	chr1:41119852–41120136	0.078 ± 0.099

**Table 4:** CpG islands whose within-pair difference in methylation rates were wide in both men and women

CpG Islands	RefGene
chr19:15833733–15833983	
chr20:54824312–54824584	MC3R
chr13:112627428–112627642	
chr19:5074591–5074814	KDM4B
chr11:67052394–67053110	ADRBK1
chr6:27482888–27483089	
chr6:139116946–139117469	CCDC28A
chr9:135361992–135362481	C9orf171
chr1:149162389–149162615	
chr1:75590817–75591354	LHX8
chr12:125003217–125003482	NCOR2

*RefGene* Reference gene mainly according to UCSC database

### Gender difference index of WPDMs

As shown in Additional file 3: Figure S2, the gender difference indices of WPDMs were positive for 86.0 % (21439/24932) of autosomal CpG islands, but negative for 76.7 % (556/725) of X-chromosomal CpG islands.

### Comparison of each WPDM between older male and female pairs

**Fig. 2:**
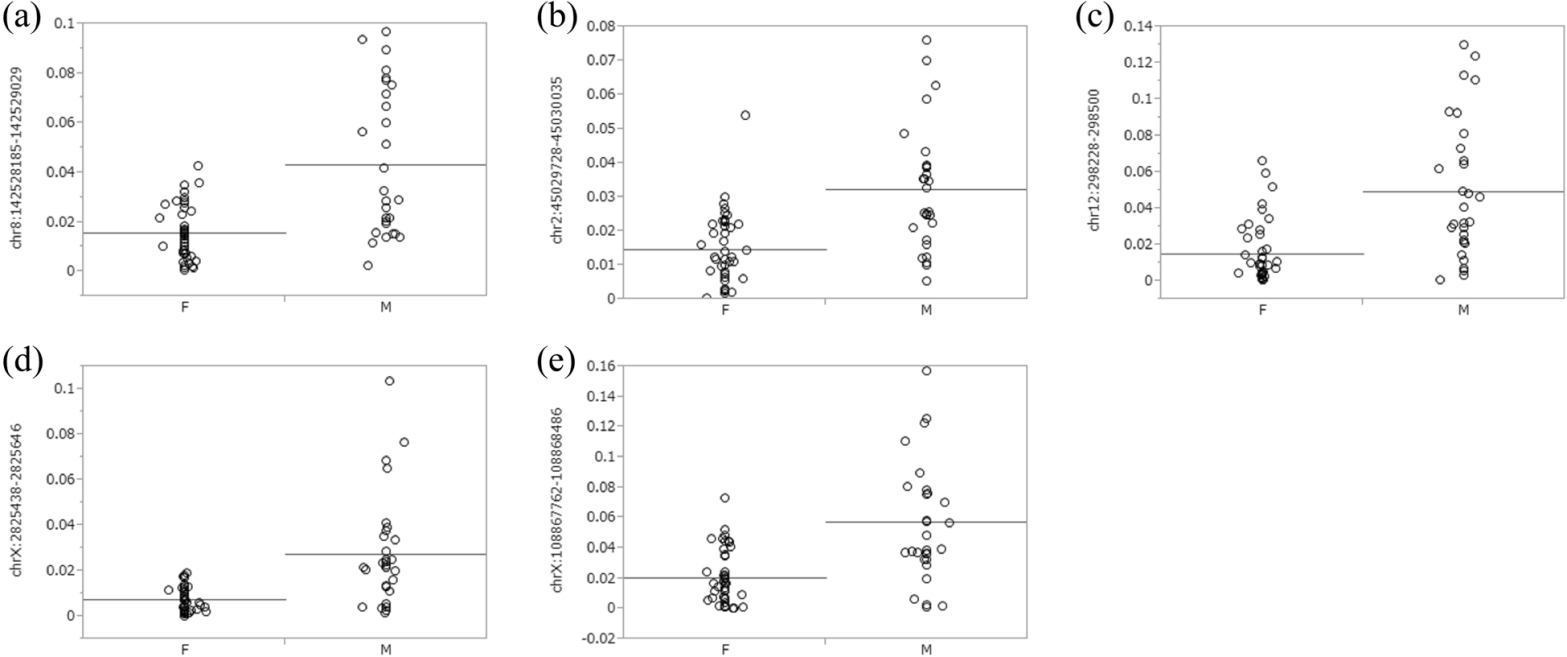
CpG islands showing greater within-pair differences in methylation levels for older male pairs. See "Scatter chart" column of Table 5 for explanation of each panel

**Fig. 3:**
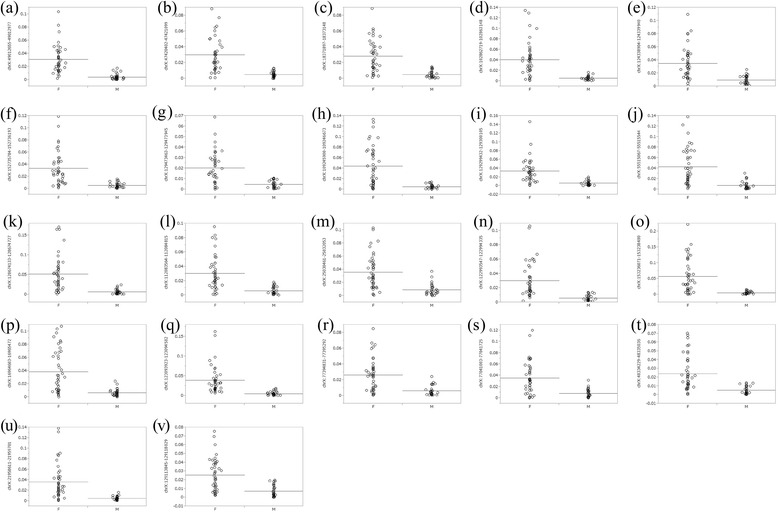
CpG islands showing greater within-pair differences in methylation levels for older female pairs. See "Scatter chart" column of Table 5 for explanation of each panel

Of the 25657 CpG islands analyzed, 11461 CpG islands showed low P values (<0.05) for WPDMs between male and female pairs using Student’s *t* test. Among these significant CpG islands, WPDMs in the male pairs were higher in 11027 CpG islands (10975 were autosomal and 52 were X chromosome), whereas those in female pairs were higher in the other 434 islands (51 were autosomal and 383 were X chromosome) (Additional file 4: Table S2). To perform multiple comparisons, we corrected the P values using the Bonferroni method and found 27 significant CpG islands. Of them, 3 were in autosomal chromosomes (2, 8, 12 chromosomes) and 24 were in the X chromosomes (Table 5). The WPDM in male pairs was significantly higher in all 3 autosomal CpG islands (Fig. 2a–c) and 2 of 24 X chromosomal island (Figs. 2d, 2e). Those in the female pairs were significantly higher in 22 of 24 X chromosomal CpG islands (Figs. 3a-v).

**Table 5:**
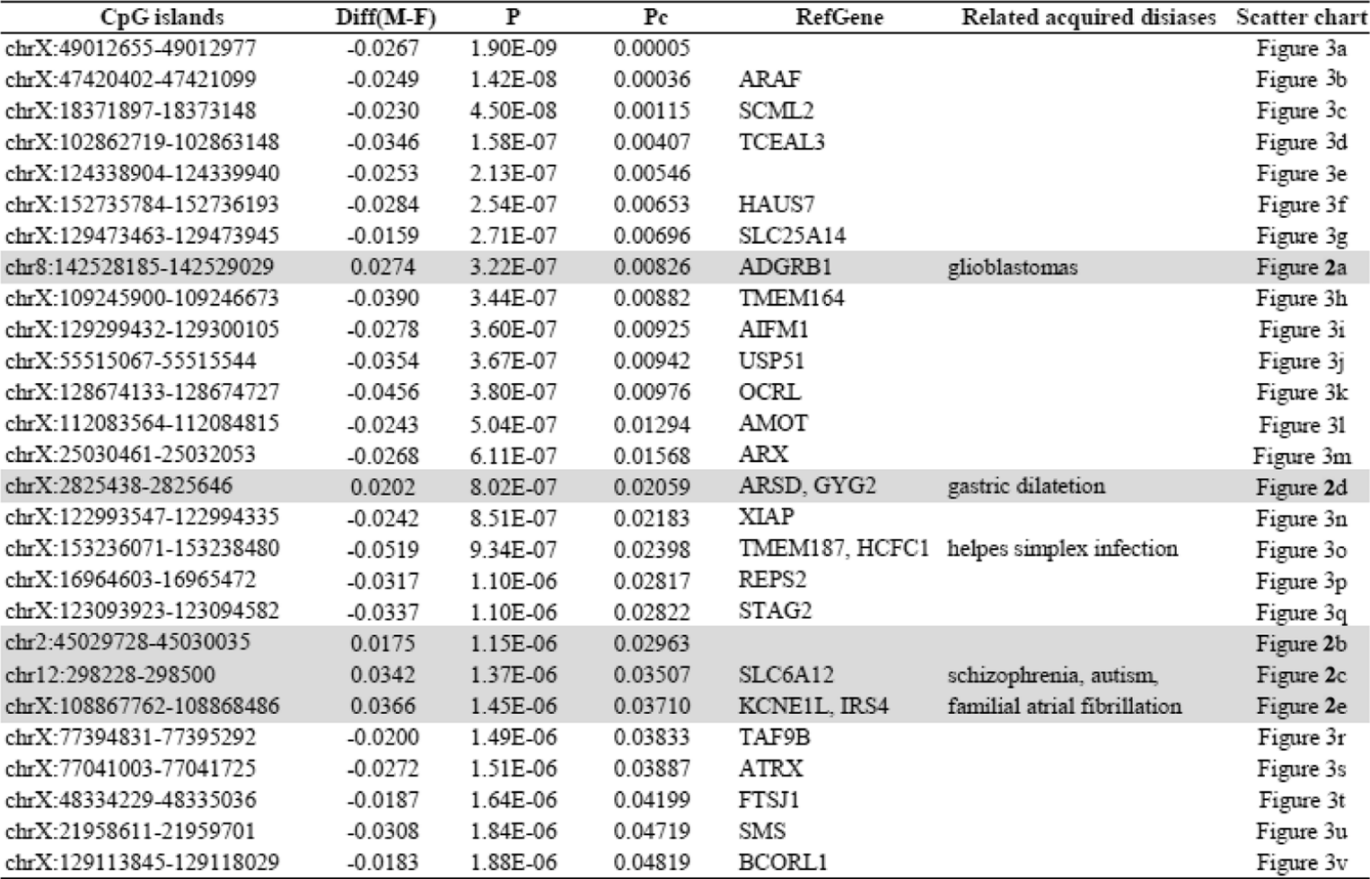
CpG islands with signigicant difference in WPDMs between men and women pairs

Diff (M-F): The difference of mean WPDM between men and women pairs. Mean WPDM of shaded CpG islands were higher in male pairs. Pc: Corrected P using Bonferroni method. RefGene: Reference gene mainly according to UCSC database. WPDM in each pair is shown in the appropriate figures

## Discussion

We clarified in this study that some CpG islands show large WPDMs both in men and women (Table 4), WPDMs of autosomal CpG islands are generally large in men and those of X-chromosomal CpG islands are generally large in women (Fig. 1, Additional file 1: Figure S1 and Table 2), and multiple comparison indicated the significant differences in WPDMs of some CpG islands between men and women (Table 5) (Figs. 2 and 3). We suppose that these may be caused by the sex-related differences in sensitivity to the DNA methylation or the sex-related difference in the exposure to environment. Therefore, it will be required extra attention to sex-related individual differences when we analyze DNA methylation.

According to the UCSC database [22, 23], the CpG islands with large WPDMs common to both male and female pairs (Table 4) are located near the genes encoding the MC3R (melanocortin 3 receptor), KDM4B (lysine (K)-specific demethylase 4B), *ADRBK1* (adrenergic beta receptor kinase 1, also known as GRK2), CCDC28A (coiled-coil domain containing 28A), C9orf171 (chromosome 9 open reading frame 171), LHX8 (LIM homeobox 8), NCOR2 (nuclear receptor corepressor 2), and so on (Table 4). Two of the genes, MC3R and ADRBK1, are related to the regulation of energy homeostasis [24, 25]. Such genes may be susceptible epigenetic changes by environmental factors in both men and women. In addition, these results will serve the data as controls when interpreting the biological relevance of sex-related CpG islands.

In the present study, we found that the WPDMs of most X chromosomal CpG islands are larger in female pairs. This may be due to the random inactivation of the X chromosome, which is specific for females [26]. Interestingly, the WPDMs of most autosomal CpG islands were larger in male pairs. We confirmed these data using older twins because the WPDMs increase with age [27–30]. These indicate that individual differences in most autosomal methylation levels are greater in men than women and suggest that epigenetic changes of DNA in autosomal chromosomes may be more dynamic in men, indicating that men may be more sensitive to environmental factors or may encounter more opportunities to interact with environmental factors compared to women.

It is possible that the large differences in WPDMs of particular gene between men and women may be related to the sex differences in the disease susceptibility of acquired diseases which affected by DNA methylation in that gene. In the present study, statistical analyses indicate that WPDMs were significantly greater in 3 autosomal (Figs. 2a-c) and 2 X chromosomal CpG islands in men (Figs. 2d and e), but were significantly greater in 22 X chromosomal CpG islands in women (Figs. 3a-v). Two of these autosomal CpG islands are located near known genes, *ADGRB1* (adhesion G protein-coupled receptor B1) and *SLC6A12* (solute carrier family 6 (neurotransmitter transporter) member 12) (Table 5). Interestingly, glioblastoma [31], gastric cancer [32], and colorectal cancer [33], which are dominant in males [34–36], are associated with *ADGRB1*, and schizophrenia [37] and autism [38], which are also dominant in males [39, 40], are associated with *SLC6A12*.

By contrast, although the WPDMs of the majority of CpG islands in the X chromosome are greater in women, the WPDMs of the two CpG islands in the X chromosome were significantly greater in male pairs. These CpG islands are located near known genes, including *ARSD* (arylsulfatase D), *KCNE1L* known as *KCNE5* (potassium channel voltage gated subfamily E regulatory beta subunit 5), *GYG2* (glycogenin 2), and *IRS4* (insulin receptor substrate 4) (Table 5). *KCNE1L* and *ARSD* are associated with atrial fibrillation [41] and gastric dilatation [42], respectively, both of which are also male dominant [43, 44]. *GYG2* is involved in blood glucose homeostasis [45] and *IRS4* encodes the insulin receptor substrate. The CpG sites in such glucose-related genes may be easily influenced by glucose levels, which are higher in men than in women [46]. On the other hand, *HCFC1* (host cell factor C1), which has a higher WPDMs in women, is associated with herpes simplex infection [47], which is female dominant [48].

Because one of the limitations of this study may be the sample size, which is not enough for high statistical power, there may be some other minor significances we could not find. Another limitation may be a lack of replication study because it is difficult to collect healthy twin data for another cohort. It will be important to analyze the age as co-factor to explore whether the pattern of sex difference changes with age although we could not because of the small sample size. In future, when DNA methylation levels are used as new laboratory tests, our data will be important to know the physiological difference and may also supply significances for diagnosis or prognosis of some sex-related disorders.

## Conclusion

In conclusion, sex-related differences were present in the WPDMs of autosomal and X-chromosomal CpG islands, which were greater in men and women, respectively for individuals with the same genetic background. These differences may be associated with the sexual influences in susceptibility of some diseases.

## Additional files

Additional file 1: Figure S1. Within-pair differences for the methylation levels (WPDMs) of each CpG island. Red circles indicate male pairs, and blue circles indicate female pairs. Within-pair differences in male pairs are greater in most autosomal CpG islands. (TIF 2145 kb) Additional file 2: Table S1. Rank order within-pair differences in methylation levels (WPDMs) of CpG islands in older male and female pairs (in descending order). (XLSX 2065 kb) Additional file 3: Figure S2. Comparison of within-pair differences in methylation levels (WPDMs) between older male and older female pairs. The gender difference index is positive when the mean within-pair differences in the methylation levels are higher in male pairs than female pairs. (TIF 298 kb) Additional file 4: Table S2. Results of statistical test comparing within-pair difference of methylation levels (WPDMs) between older male and female twin pairs. WPDMs in the male pairs were higher in 11027 CpG islands (10975 were autosomal and 52 were X chromosome), whereas those in female pairs were higher in the other 434 islands (51 were autosomal and 383 were X chromosome). (XLSX 1758 kb) Additional file 5: Table S3. Data supporting the findings in this study. (XLSX 6571 kb)

## Abbreviations

CpG: Cytosine-phosphodiester bond-Guanine
ML: Methylation level
STR: Short tandem repeat
WPDM: Within-pair differences of the methylation level
