# Supplementary figures and images for: Within-pair differences of DNA methylation levels between monozygotic twins are different between male and female pairs
Source: BMC Med Genomics. 2016 Aug 26;9(1):55. doi: 10.1186/s12920-016-0217-2 (PMC5000519; doi:10.1186/s12920-016-0217-2)

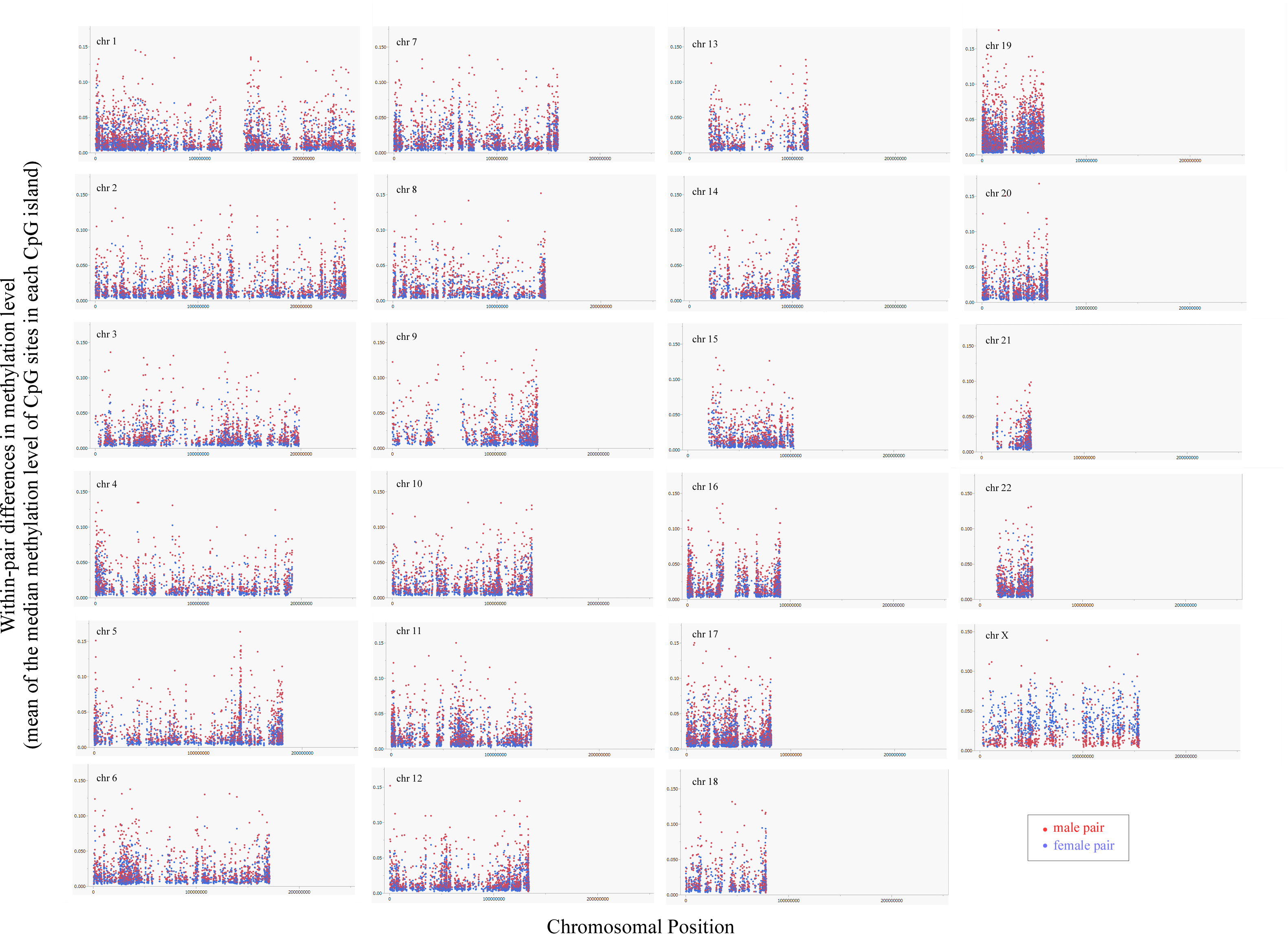

Supplement: Additional file 1: Figure S1. — Within-pair differences for the methylation levels (WPDMs) of each CpG island. Red circles indicate male pairs, and blue circles indicate female pairs. Within-pair differences in male pairs are greater in most autosomal CpG islands. (TIF 2145 kb) [file 12920_2016_217_MOESM1_ESM.tif]
